# A comparison between hydrogel spacer and endorectal balloon: An analysis of intrafraction prostate motion during proton therapy

**DOI:** 10.1002/acm2.12051

**Published:** 2017-02-25

**Authors:** Samantha G. Hedrick, Marcio Fagundes, Ben Robison, Marc Blakey, Jackson Renegar, Mark Artz, Niek Schreuder

**Affiliations:** ^1^ Provision Center for Proton Therapy Knoxville TN USA

**Keywords:** endorectal balloon, hydrogel, motion, prostate, proton therapy, SpaceOAR

## Abstract

The purpose of this study was to evaluate intrafraction prostate motion in patients treated with proton therapy and an endorectal balloon or a hydrogel spacer using orthogonal x‐rays acquired before and after each treatment field. This study evaluated 10 patients (662 fields throughout treatment) treated daily with an endorectal balloon (ERB) and 16 patients (840 fields throughout treatment) treated with a hydrogel spacer (GEL) without an ERB. Patient shifts were recorded before and after each treatment field, correlated with a treatment time, using x‐ray imaging and implanted fiducial alignment. For each shift, recorded in X, Y, and Z, a 3D vector was calculated to determine the positional change. There was a statistically significant difference in the mean vector shift between ERB (0.06 cm) and GEL (0.09 cm), (*P* < 0.001). The mean includes a large number of zero shifts, but the smallest non‐zero shift recorded was 0.2 cm. The largest shifts were, on average, in the Z direction (anterior to posterior). The average Z shift was +0.02 cm for both ERB and GEL. There was no statistical difference between ERB and GEL for shifts greater than 0.3 cm (*P* = 0.13) or greater than 0.5 cm (*P* = 0.36). For treatment times between 5 and 9 min, a majority of shifts were less than 0.2 cm, 85.9% for ERB and 73.2% for GEL. There was a significant positive correlation between the vector shifts and field time for both ERB (*r* = 0.2, *P* < 0.001) and GEL (*r* = 0.07, *P* < 0.04). We have shown that prostate motion is clinically comparable between an ERB and a hydrogel spacer, and the time dependencies are similar. A large majority of shifts for both ERB and hydrogel are well within a typical robust planning margin. For GEL patients, we chose to maintain slightly larger planning margins than for ERB due to already improved rectal sparing with GEL.

## 1 Introduction

Daily intrafraction prostate motion has always been a concern in modern radiation therapy, regardless of the treatment modality. Prostate motion is typically a function of bladder and rectal filling, and will vary day‐to‐day and during treatment. Most commonly, patients are aligned using implanted fiducials in the prostate. These fiducials can be aligned using orthogonal x‐rays and checked periodically throughout daily treatment, to evaluate prostate motion. In proton therapy, prostate cancer is traditionally treated utilizing an endorectal balloon. The benefit of an endorectal balloon is improved prostate stability and consistent rectal filling. There are several studies validating prostate motion with an endorectal balloon.^1^, ^2^, ^3^ The disadvantage of an endorectal balloon is that they can distend the rectum toward the prostate, potentially increasing the rectum volume within the treated area.

In April of 2015, a hydrogel spacer, SpaceOAR (Augmenix, Inc, Waltham, MA, USA) was approved by FDA for use in prostate radiation treatment. Since that time, this gel spacer has been utilized in several treatment modalities, including proton therapy. The hydrogel spacer is implanted between the prostate and the anterior wall of the rectum, providing additional separation and potentially improving high dose rectal sparing.^4^ The gel spacer is typically not used with an endorectal balloon, so the traditional balloon motion data is not necessarily applicable.

It is clinically important to understand the effect of a hydrogel spacer and empty rectum on prostate motion during treatment, because this motion defines planning target margins and image guided radiation therapy (IGRT) protocols. Modern radiation therapy is moving toward hypofractionation and smaller PTV margins.^5^, ^6^ Increased intrafraction motion could be detrimental in moving toward this goal. Increased intrafraction motion would also likely require increased imaging during treatment, thus increasing patient treatment times and decreasing overall efficiency. Additionally, increased prostate motion could lead to uncertainties in the treatment and potentially misrepresent the true bladder and rectal sparing.

The effect of a hydrogel spacer on prostate motion has been studied previously, comparing patients treated with gel and with an empty rectum without gel. One study utilized Calypso and found that the intrafraction motion is unaffected by the presence of the hydrogel.^7^ Another study utilized cone beam CT (CBCT) to track interfraction motion.^8^ They found that prostate displacements greater than 0.5 cm were similar for patients treated with and without a hydrogel spacer, confirming that the prostate is not destabilized by the presence of the hydrogel. To date, a motion study comparing a hydrogel spacer versus the use of an endorectal balloon has not been conducted. Because an endorectal balloon can distend the rectum, we chose not to use an endorectal balloon in conjunction with the hydrogel spacer.

The purpose of this study, therefore, was to evaluate intrafraction motion of the prostate in patients treated with proton therapy and an endorectal balloon or a hydrogel spacer implant using orthogonal x‐rays acquired before and after each treatment field.

## 2 Methods

This study evaluated 26 patients; 10 of whom were treated daily with an endorectal balloon (ERB) and 16 were treated with hydrogel spacer implant (GEL) without an ERB.

### 2.A Fiducial and hydrogel spacer implant

Our fiducial and hydrogel implant procedure has been described elsewhere and is summarized, as follows.^9^ All fiducial marker placements were performed during the same outpatient procedure under local perineum skin numbing, following general application technique guidelines previously published. Specifically, at our facility, all patients had a fleet enema 2–3 hours prior to the procedure. For each patient, three fiducial markers were implanted: one in the right posterior base, one in the right posterior apex, and one placed in the left anterior midgland.

A single radiation oncologist performed all hydrogel and fiducial placements. There were no instances of rectal needle penetration and all procedures were performed without complications.

### 2.B Treatment planning

On the day of the treatment planning CT (TPCT), patients were instructed to drink a certain volume of water at a known time before the TPCT, to ensure bladder fullness and reproducibility. All GEL patients were instructed to perform a fleet enema the morning of the TPCT. ERB patients had a 60 cc RadiaDyne endorectal balloon placed in their rectum and the balloon was filled with water. All patients were simulated supine, with a vacuum bag and knee immobilization. All patients also underwent MRI scans on the same day as the TPCT for improved target visualization. MRI scans were also useful for visualizing the hydrogel spacer.

The TPCT and MRIs were fused, based on fiducials, to allow for target and hydrogel contouring on the TPCT. For all patients, the clinical target volume (CTV) for low risk patients included only the prostate, as visualized on MRI and CT fusion; the CTV for intermediate risk included the proximal and medial 1 cm of the seminal vesicles on the first phase of the treatment with a subsequent boost to CTV2, defined as the prostate. For ERB patients, the planning target volume (PTV) is an expansion of the CTV, 0.3 cm posteriorly and 0.4 cm elsewhere. For GEL patients, the planning target volume (PTV) is an expansion of the CTV, 0.5 cm posteriorly and 0.6 cm elsewhere. Increased margins were utilized for GEL patients due to the lack of a priori motion data. Additionally, because the rectum was displaced away from the prostate due to the hydrogel implant, larger margins did not necessarily provide additional high dose to the rectum. In addition, the PTV Evaluation (PTV_Eval) structure is an expansion of the PTV, 0.5 cm in the direction of the beams. For each plan in this analysis, lateral beams were utilized. PTV_Eval was used for inverse planning to increase dose range laterally, which improves dose coverage and overall plan robustness.

**Figure 1:**
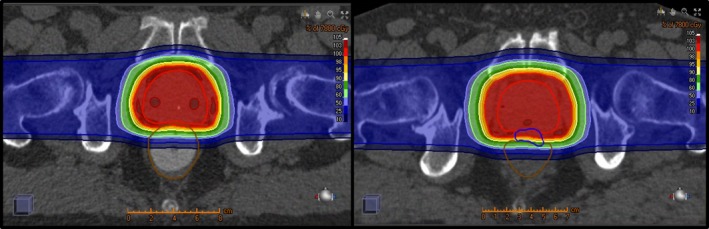
Left: CT of a prostate patient with an endorectal balloon (ERB). Right: CT of a prostate patient with a hydrogel spacer (GEL). Dose shown to a prescription of 78 Gy_RBE_.

Treatment was planned using the RayStation planning system with the following objectives and dose constraints: For target coverage, 95% of the PTV was to receive 100% of the prescribed dose and 100% of the PTV was planned to receive a minimum of 95% of the prescription dose. Generally, 99% of the CTV received 100% of the prescription dose. We have previously documented the ability to reach a rectum V90% of ≤1% using pencil beam scanning proton therapy and SpaceOAR with the margins and expansions described above. Therefore, our rectum OAR constraint was routinely set at V90% ≤1% for GEL patients and ≤10% for ERB patients, while maintaining target coverage priority. Each patient in this study was treated with two opposing lateral fields. Each patient underwent a robust evaluation by a medical physicist, analyzing the effects of patient motion in all directions, 3 degrees of roll, 3 degrees of yaw, and 2.5% + 0.1 cm range uncertainty. For ERB patients, the patient was shifted 0.3 cm in all directions, GEL patients were shifted 0.5 cm in all directions. Under all perturbations, the prostate CTV must maintain V100% ≥95%. Typical dose distributions are shown for both ERB and GEL in Fig. 1.

### 2.C Image guidance

For daily treatment, the patient was set up in the treatment position based on lasers and patient tattoos. The patient was then imaged using two orthogonal x‐rays. On the DRR, each fiducial had a 0.2 cm expansion created, known in‐house as the “grape”. In this study, the patient's shifts were recorded before and after each treatment field, correlated with a treatment time, in order to determine the amount of motion within each treatment time. This data was assessed using x‐ray imaging and fiducial alignment. Therapists were instructed to record the amount of shift necessary, in X, Y, and Z, to return each fiducial to within its respective “grape”. In order to treat, all 3 of the patient's fiducials must align within the respective grape. The shifts prior to field 1 were not recorded and were considered to be the zero point. Therapists did record the time at which the first images were acquired.

**Figure 2:**
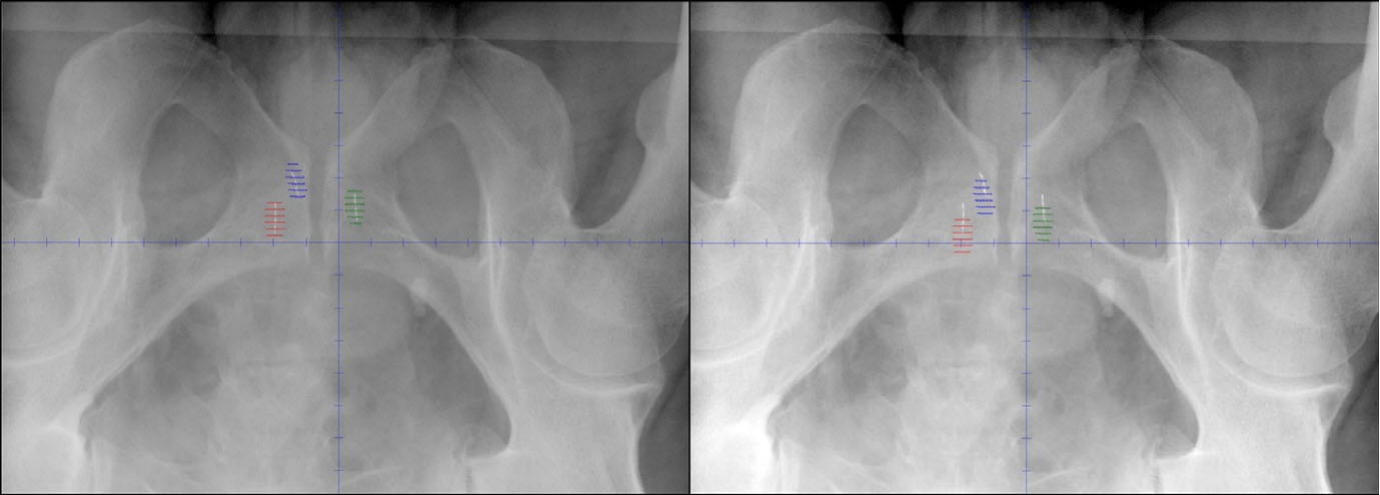
X‐ray image with overlaid fiducial expansion contours (grapes). Left: Image acquired before a treatment, with fiducials aligned within grapes. Right: Image acquired after treatment, fiducials are slightly shifted.

Following the patient's first treatment field, the patient was imaged again. Both pre‐ and post field images are shown in Fig. 2, illustrating fiducials inside the grapes for a pre‐field image and a typical shift seen on a post field image. Therapists determined the amount of shift necessary to return the fiducials to within the grapes and these shifts were recorded. Therapists also recorded the time at which the images were acquired. These are the “Post field 1” shifts and time. Although the necessary shifts were recorded, the table was not translated at this time.

At our center, we have two treatment rooms with a gantry and one treatment room with a fixed beam. In the gantries, the gantry was rotated 180 degrees between treatment fields. For the fixed beam room, the treatment table was rotated 180 degrees between fields. Following gantry or table rotation, the patient was imaged again and the necessary shifts and time point were recorded. These were the “Pre Field 2” shifts and time. At this point, the table was translated to return the fiducials to the center of the grapes. The second field was delivered and the patient was imaged a final time. These were the “Post field 2” shifts and time. The necessary shifts were recorded but not applied. For all images, if the fiducials were still in the grapes, even marginally, a zero shift was recorded.

### 2.D Motion analysis

The patients were separated in to ERB and GEL, to evaluate the difference in intrafraction motion. For each fraction, the time during field 1 and field 2 were calculated. The intrafraction motion for each field was defined as the shift values recorded for the post field images. The Pre Field 1 shifts were assumed to be zero and not recorded. The Pre Field 2 shifts were recorded but not analyzed. Because the fiducials were aligned within the grapes before each treatment field, the post field shifts indicate the motion that occurred during each field. For each shift, recorded in X, Y, and Z, a resultant 3D vector was calculated to determine the 3D positional change. This is referred to as the vector analysis. We also evaluated the average changes in X, Y, and Z, referred to as the directional analysis. This allowed us to determine if one group showed prevalence in one shift direction over another. Finally, we were interested in the correlation of time with shift magnitude, e.g., does a longer treatment time lead to larger shifts.

The vector shift distributions were not normally distributed, so they were analyzed using non‐parametric statistics with medians and interquartile ranges. Due to a non‐normal distribution, the time correlation was analyzed using non‐parametric Spearman's rho correlations.

## 3 Results

### 3.A Vector analysis

**Figure 3:**
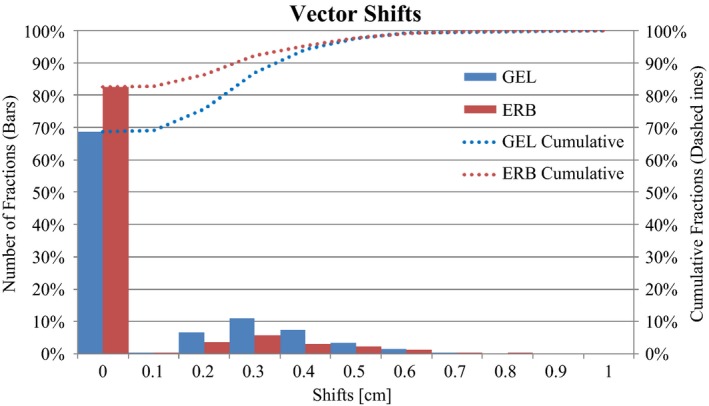
Histogram of vector shift distribution for ERB (red) and GEL (blue). Dashed lines indicate cumulative fractions. Due to the 0.2 cm fiducial expansion, shifts less than 0.2 cm were recorded as zero shifts.

Ten ERB patients were analyzed for a total of 662 fields. Sixteen GEL patients were analyzed for a total of 840 fields. There was a statistically significant difference in the mean vector shift between ERB (0.06 cm) and GEL (0.09 cm), (*P* < 0.001). Figure 3 illustrates the distribution of vector shifts for ERB and GEL.

It should be noted that the mean shift includes a large number of zero shifts (83% for ERB and 69% for GEL). The smallest non‐zero shift recorded was 0.2 cm, which is the threshold distance of the “grape”.

### 3.B Directional analysis

The largest shifts were, on average, in the Z direction (anterior to posterior). The average Z shift was +0.02 cm (anterior) for both ERB and GEL when evaluating both positive and negative shift values. When evaluating the absolute values of the shifts, Z remained the largest shift, at 0.03 cm for ERB and 0.06 cm for GEL.

Table 1 compiles the summation data for the vector shifts and the directional shifts. 97.7% of the ERB patients and 97.5% of the GEL patients had a vector shift that was less than or equal to our GEL robust evaluation threshold of 0.5 cm.

**Table 1:** Cumulative fractions for each mm of shift greater than the fiducial expansion (0.2 cm) for vector and directional shifts

Shifts (cm)	3D		X		Y		Z	
	GEL	ERB	GEL	ERB	GEL	ERB	GEL	ERB
≤0.2	75.8%	86.4%	97.1%	96.1%	90.6%	93.8%	84.9%	91.5%
≤0.3	86.8%	92.2%	99.2%	99.1%	95.5%	97.9%	96.0%	97.0%
≤0.4	94.2%	95.3%	99.4%	99.5%	98.3%	98.8%	98.5%	98.2%
≤0.5	97.5%	97.7%	99.8%	100.0%	99.5%	99.8%	99.5%	99.5%
≤0.6	99.1%	99.1%	100.0%	100.0%	99.9%	99.8%	99.6%	99.8%
≤0.7	99.5%	99.5%	100.0%	100.0%	100.0%	99.8%	99.9%	100.0%
≤0.8	99.6%	99.8%	100.0%	100.0%	100.0%	100.0%	100.0%	100.0%
≤0.9	99.9%	100.0%	100.0%	100.0%	100.0%	100.0%	100.0%	100.0%
≤1	100.0%	100.0%	100.0%	100.0%	100.0%	100.0%	100.0%	100.0%

**Figure 4:**
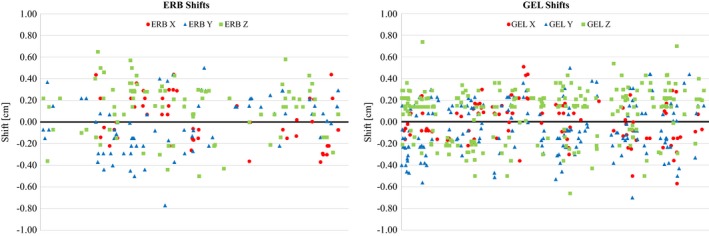
Scatter plot of directional shifts. Left: ERB. Right: GEL. Circles: X. Triangles: Y. Squares: Z.

A scatter plot was also created for the individual shifts, evaluating X, Y, and Z, shown in Fig. 4. All zero shifts were excluded from the graph for illustrative purposes.

### 3.C Probability of shifts

During this study, we used a robust evaluation tolerance of 0.5 cm for GEL, therefore, we chose to evaluate patients who had a vector shift or directional shift that exceeded this value. The total number of fields with a vector shift exceeding 0.5 cm was 19 of 662 fields for ERB (2.9%) and 21 of 840 fields for GEL (2.5%).

We also evaluated the vector shift distribution difference between ERB and GEL for vector shifts greater than 0.3 cm (ERB robust) and greater than 0.5 cm (GEL robust). There was no statistical difference between ERB and GEL for shifts greater than 0.3 cm (*P* = 0.13) or greater than 0.5 cm (*P* = 0.36).

When evaluating directional shifts greater than 0.5 cm, ERB patients had 9 shifts in X, Y, or Z in 9 of 662 fields (1.4%), i.e., unidirectional shifts. In GEL patients, shifts in more than one direction within the same field were identified in three fields, for a total of 16 shifts in 13 of 840 fields (1.5%).

### 3.D Effect of time

Field 1 was evaluated separately from field 2 and it was found that, on average for ERB, field 1 had a longer time (8 min for field 1, 5 min for field 2) and larger average vector shift (0.07 cm for field 1, 0.04 cm for field 2). For GEL, field 1 had a longer treatment time (6 min for field 1, 4 min for field 2) and the average vector shift was the same (0.09 cm). The longer field 1 time is due to the time required for initial alignment of the patient. On a daily basis, therapists first align the patient using tattoos and lasers. Field 1 will typically require more images and longer imaging time to move the patient from the initial alignment point to the treatment location. Ideally, the final image before treating field 1 would have been used as the Pre Field 1 time, but for convenience and consistency, therapists recorded the time of the first image for each field. For field 2, the patient is already within a few millimeters of the treatment location, so the imaging time is shorter.

**Figure 5:**
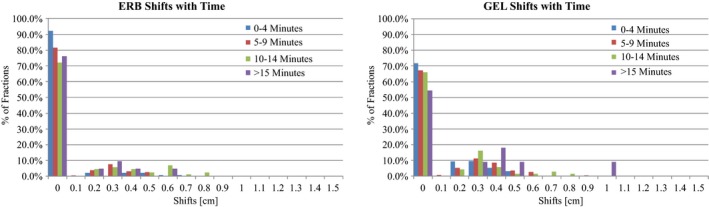
Histogram of vector shift distribution with respect to time. Time is broken into 4 time points, 0–4 min (blue), 5–9 min (red), 10–14 min (green), and greater than 15 min (purple). Left: ERB. Right: GEL. Due to the 0.2 cm fiducial expansion, shifts less than 0.2 cm were recorded as zero shifts.

The average time per field was 7 min for ERB and 5 min for GEL. For each fraction, the time per field correlated with a vector shift. For ERB and GEL, a majority of the field times were between 5 and 9 min. In this time period, a majority of the shifts were less than 0.2 cm, 85.9% for ERB and 73.2% for GEL. There was a significant positive correlation between the vector shifts and field time for both ERB (*r* = 0.2, *P* < 0.001) and GEL (*r* = 0.07, *P* < 0.04). A histogram of the distribution of shifts with respect to time is shown in Fig. 5 for both ERB and GEL. In Table 2, the number of fields and mean vector shift for each time block is shown for ERB and GEL.

**Table 2:** Mean vector shift comparison between ERB and GEL for each time point with corresponding p value

Time (min)	Data group	Number of fields	Mean vector shift (cm)	*P* value
0–4	GEL	312	0.07	<0.001
	ERB	143	0.03	
5–9	GEL	414	0.10	<0.001
	ERB	390	0.05	
10–14	GEL	68	0.11	0.70
	ERB	86	0.12	
>15	GEL	11	0.21	0.13
	ERB	21	0.07	

## 4 Discussion

This study identified a mean vector shift of 0.06 cm for ERB and 0.09 cm for GEL. While this is statistically different, it is not clinically relevant since both values are less than the minimum robust evaluation tolerance of 0.3 cm. When evaluating shifts greater than 0.3 cm and greater than 0.5 cm, there is no longer a statistical difference in the vector shifts between ERB and GEL. On rare occasions, there were shifts greater than 0.5 cm for both ERB and GEL patients, which indicates that the need for imaging between treatment fields remains. Our data has shown, however, that the likelihood of several large shifts, i.e. greater than 0.5 cm, for a single patient is low.

For smaller shifts, less than 0.3 cm, GEL has more non‐zero shifts than ERB. This indicates that the prostate is more likely to move over a short distance with GEL than with ERB. Based on this data, we felt comfortable reducing our posterior PTV expansion margin from 0.5 cm to 0.4 cm for GEL patients. However, we chose to maintain the 0.6 cm GEL expansion in all other directions since it does not include biologically critical tissues. Additionally, because the GEL is proven to improve rectal sparing over ERB, we did not feel the need to reduce the posterior margins to match the 0.3 cm ERB expansion. Even with a larger posterior PTV expansion, the GEL rectal sparing is still superior to ERB.^10^

For both ERB and GEL, there is a tendency to have increased shifts with increased treatment time. A majority of our patients, for both ERB and GEL, were treated within 5–9 min. Within this time block, small GEL shifts were statistically higher than ERB shifts. However, similar to the overall shifts, the values were statistically relevant, but not necessarily clinically relevant. The ERB mean shift was 0.03 cm and the GEL mean shift was 0.07 cm. While the GEL shift magnitude is twice as large as ERB, the shifts are sub‐millimeter and well within the robust tolerance. For times greater than the average, there is no difference between GEL and ERB. This indicates that the effect of rectal filling does not seem to have a significant effect on prostate motion during the treatment time.

## 5 Conclusions

For patients undergoing proton radiation therapy for prostate cancer, prostate motion with a hydrogel spacer is clinically equivalent to motion with an endorectal balloon. We have shown that the mean shifts are clinically comparable between an endorectal balloon and a hydrogel spacer, and the time dependencies are similar. A large majority of shifts for both balloon and hydrogel are well within a typical robust CTV margin. For GEL patients, we chose to maintain slightly larger planning margins than for ERB due to already improved rectal sparing with hydrogel.

## Conflict of Interest

Authors have no conflicts of interest to disclose.

## References

[1] Joo JH , Kim YJ , Kim YS , et al. Analysis of prostate bed motion using an endorectal balloon and cone beam computed tomography during post prostatectomy radiotherapy. OncoTarget and Therapy. 2016;9:3095–3100. 10.2147/OTT.S98112 PMC4888733 27307750

[2] Both S , Wang KK‐H , Plastaras JP , et al. Real‐time study of prostate intrafraction motion during external beam radiotherapy with daily endorectal balloon. Int J Radiat Oncol Biol Phys. 2011;81:1302–1309. 21035952 10.1016/j.ijrobp.2010.08.052

[3] Smeenk RJ , Louwe RJW , Langen KM , et al. An endorectal balloon reduces intrafraction prostate motion during radiotherapy. Int J Radiat Oncol Biol Phys. 2012;832:661–669. 10.1016/j.ijrobp.2011.07.028 22099035

[4] Gysen K , Kneebone A , Alfieri F , Guo L , Eade T . Feasibility of and rectal dosimetry improvement with the use of SpaceOAR^®^ hydrogel for dose‐escalated prostate cancer radiotherapy. J Med Imaging Radiat Oncol. 2014;58:511–516. 24580893 10.1111/1754-9485.12152

[5] Madsen BL , Hsi RA , Pham HT , et al. Stereotactic hypofractionated accurate radiotherapy of the prostate (SHARP), 33.5 Gy in five fractions for localized disease: First clinical trial results. Int J Radiat Oncol Biol Phys. 2007;67:1099–1105. 17336216 10.1016/j.ijrobp.2006.10.050

[6] Kupelian PA , Willoughby TR , Reddy CA , et al. Hypofractionated intensity‐modulated radiotherapy (70 gy at 2.5 Gy per fraction) for localized prostate cancer: Long‐term outcomes. Int J Radiat Oncol Biol Phys. 2005;63:1463–1468. 16169683 10.1016/j.ijrobp.2005.05.054

[7] Juneja P , Kneebone A , Booth JT , et al. Prostate motion during radiotherapy of prostate cancer patients with and with application of a hydrogel spacer: A comparative study. Radiation Oncology. 2015;10:215. 26499473 10.1186/s13014-015-0526-1 PMC4619294

[8] Picardi C , Rouzaud M , Kountouri M , et al. Impact of hydrogel spacer injections on interfraction prostate motion during prostate cancer radiotherapy. Acta Oncol. 2016;55:834–838. 26796870 10.3109/0284186X.2015.1128118

[9] Hedrick S , Fagundes M , Case S , et al. Validation of rectal sparing throughout course of treatment in prostate cancer patients treated with SpaceOAR. J Appl Clin Med Phys. 2017;18:82–89. 28291933 10.1002/acm2.12010 PMC5689883

[10] Fagundes M , Hedrick S , Robison B , et al. Evolving rectal sparing in fiducial based image guided proton therapy for localized prostate cancer. Int J Radiat Oncol Biol Phys. 2016;96.2S:E279.

